# Transnational Tobacco Company Interests in Smokeless Tobacco in Europe: Analysis of Internal Industry Documents and Contemporary Industry Materials

**DOI:** 10.1371/journal.pmed.1001506

**Published:** 2013-09-10

**Authors:** Silvy Peeters, Anna B. Gilmore

**Affiliations:** Department for Health, University of Bath, Bath, United Kingdom; San Diego State University, United States of America

## Abstract

**Background:**

European Union (EU) legislation bans the sale of snus, a smokeless tobacco (SLT) which is considerably less harmful than smoking, in all EU countries other than Sweden. To inform the current review of this legislation, this paper aims to explore transnational tobacco company (TTC) interests in SLT and pure nicotine in Europe from the 1970s to the present, comparing them with TTCs' public claims of support for harm reduction.

**Methods and Results:**

Internal tobacco industry documents (in total 416 documents dating from 1971 to 2009), obtained via searching the online Legacy Tobacco Documents Library, were analysed using a hermeneutic approach. This library comprises documents obtained via litigation in the US and does not include documents from Imperial Tobacco, Japan Tobacco International, or Swedish Match. To help overcome this limitation and provide more recent data, we triangulated our documentary findings with contemporary documentation including TTC investor presentations. The analysis demonstrates that British American Tobacco explored SLT opportunities in Europe from 1971 driven by regulatory threats and health concerns, both likely to impact cigarette sales negatively, and the potential to create a new form of tobacco use among those no longer interested in taking up smoking. Young people were a key target. TTCs did not, however, make SLT investments until 2002, a time when EU cigarette volumes started declining, smoke-free legislation was being introduced, and public health became interested in harm reduction. All TTCs have now invested in snus (and recently in pure nicotine), yet both early and recent snus test markets appear to have failed, and little evidence was found in TTCs' corporate materials that snus is central to their business strategy.

**Conclusions:**

There is clear evidence that BAT's early interest in introducing SLT in Europe was based on the potential for creating an alternative form of tobacco use in light of declining cigarette sales and social restrictions on smoking, with young people a key target. We conclude that by investing in snus, and recently nicotine, TTCs have eliminated competition between cigarettes and lower-risk products, thus helping maintain the current market balance in favour of (highly profitable) cigarettes while ensuring TTCs' long-term future should cigarette sales decline further and profit margins be eroded.

*Please see later in the article for the Editors' Summary*

## Abstract 2

In light lobbying by transnational tobacco companies to remove the European Union ban on the sale of snus (a smokeless tobacco product), Silvy Peeters and Anna Gilmore explore the motivation behind tobacco companies' interests in smokeless tobacco products in Europe.

*Please see later in the article for the Editors' Summary*

## Editors' Summary

**Background:**

Every year, about 5 million people die from cancer, heart disease, and other tobacco-related diseases. In recent years, to reduce this growing loss of life, international and national bodies have drawn up various tobacco control conventions and directives. For example, the European Union (EU) Directives on tobacco control call for member states to ban tobacco advertising, promotion, and sponsorship and to adopt taxation policies aimed at reducing tobacco consumption. The 2001 EU Tobacco Products Directive also bans the sale of snus, a form of smokeless tobacco (SLT), in all EU countries except Sweden. Snus, which originated in Sweden in the early 19th century, is a moist tobacco product that is placed under the upper lip. Although snus is considerably less harmful than smoking, the sale of snus was banned in the EU in 1992 because of fears that it might cause cancer and was being marketed to young people. When Sweden joined the EU in 1994, exemption from the ban was made a condition of the membership treaty.

**Why Was This Study Done?:**

Transnational tobacco companies (TTCs) have been investing in European snus manufacturers since 2002 and more recently in pure nicotine products, and it has been suggested that, faced with declining cigarette markets in Europe and elsewhere, TTCs are preparing for a “post-cigarette era”. Since 2008, TTCs have been lobbying EU member states and the European Commission to remove the ban on snus sales, arguing that public health would be improved if governments allowed potentially reduced-harm products like snus onto the market. At the end of 2012, however, the European Commission proposed that the ban on snus sales should be continued. Here, to help inform this controversial policy debate, the researchers explore the interest of TTCs in SLT and pure nicotine in Europe from the 1970s to the present by examining internal tobacco documents and compare these interests with public claims of support for harm reduction made by TTCs.

**What Did the Researchers Do and Find?:**

By searching the Legacy Tobacco Documents Library (internal tobacco industry documents released following US litigation cases), the researchers identified 416 documents that detail the historical interest of TTCs in SLT and pure nicotine and their efforts to enter European markets, and to influence national and EU public-health policy. The researchers analyzed these documents using a “hermeneutic” approach—methodical reading and re-reading of the documents to identify themes and sub-themes. Finally, they used TTC investor presentations and other documents to confirm these themes and to provide recent data on TTC investment in SLT. British American Tobacco (BAT) explored the opportunities for marketing SLT products in Europe from 1971 onwards. This exploration was driven by regulatory threats and health concerns, both of which were likely to impact tobacco sales, and by the potential to create a new form of tobacco use among people no longer interested in taking up smoking. TTCs did not begin to invest in SLT, however, until 2002, a time when EU cigarette sale volumes started to decline, smoke-free legislation was being introduced, and tobacco harm reduction first became a major public-health issue. All the TTCs have now invested in snus even though snus test markets appear to have failed and even though there is little evidence in corporate materials that snus is central to the business strategy of TTCs.

**What Do These Findings Mean?:**

These findings suggest that BAT's early interest in SLT in Europe was driven by business concerns and was based on the potential for creating an alternative form of tobacco use among people—particularly young people—who would no longer take up smoking because of health concerns. They also suggest that TTC investments in snus were defensive—by buying up snus manufacturers and more recently nicotine producers, TTCs have eliminated competition between cigarettes and lower-risk products, thereby helping to maintain the current market balance in favor of cigarettes while ensuring the long-term future of TTCs should cigarette sales decline further. Although these findings are limited by the possibility that some relevant documents may have been omitted from this analysis, they nevertheless raise the concern that, if TTC investment in SLT continues, competition between cigarettes and SLT will reduce the potential for harm reduction to benefit public health. Legalization of snus sales in the European Union may therefore have considerably less benefit than envisaged.

**Additional Information:**

Please access these Web sites via the online version of this summary at http://dx.doi.org/10.1371/journal.pmed.1001506.

- The World Health Organization provides information about the dangers of tobacco (in several languages) and about the Framework Convention on Tobacco Control, an international treaty for tobacco control; for information about the tobacco industry's influence on policy, see the 2009 World Health Organization report Tobacco interference with tobacco control
- Details of European Union legislation on the manufacture, presentation, and sale of tobacco products is available (in several languages)
- Wikipedia has pages on tobacco harm reduction and on snus (note: Wikipedia is a free online encyclopedia that anyone can edit; available in several languages)
- The Legacy Tobacco Documents Library is a searchable public database of tobacco company internal documents detailing their advertising, manufacturing, marketing, sales, and scientific activities
- The UK Centre for Tobacco Control Studies is a network of UK universities that undertakes original research, policy development, advocacy, and teaching and training in the field of tobacco control
- SmokeFree, a website provided by the UK National Health Service, offers advice on quitting smoking and includes personal stories from people who have stopped smoking
- Smokefree.gov, from the US National Cancer Institute, offers online tools and resources to help people quit smoking
- TobaccoTactics.org, an online resource managed by the University of Bath, provides up-to-date information on the tobacco industry and their tactics to influence tobacco regulation

## Introduction

The European cigarette market, the second largest in the world by volume [1] and highly profitable to the transnational tobacco companies (TTCs) [2],[3], is shrinking [4]. Although the tobacco industry has hitherto successfully raised cigarette prices to offset volume declines, thereby maintaining or increasing profits [5], financial analysts have questioned the sustainability of this pricing strategy in the medium to long term [6]. It has also been suggested that the days of the traditional cigarette are numbered and that TTCs are preparing for a “post-cigarette era” [7]. These trends would appear to make alternative products an increasingly attractive option for TTCs who have been investing in European manufacturers of snus (a smokeless tobacco [SLT] product), and more recently in pure nicotine products [8]. Furthermore, British American Tobacco (BAT), Imperial Tobacco, Japan Tobacco International (JTI), and Phillip Morris International (PMI) have all recently become members, and sit on the Board of Directors, of the European Smokeless Tobacco Council (ESTOC), a pan-European SLT lobby group established in 1989 [9],[10].

Despite these TTC investments, and unlike many other parts of the world, SLT use is not well established in European Union (EU) markets and an estimated 92% of revenue in the global tobacco market is still generated from cigarettes [11]. According to the 2011 European survey for Smoking [12], *regular* use of SLT is significant only in Sweden at 11%, compared to 2% or less in other EU Member States. This reflects the fact that sales of certain forms of SLT, notably snus, have been prohibited in EU countries other than Sweden since 1992 [13]. Regular snus use is also significant in Norway, a European Economic Area (but not EU) member state, where daily snus use increased from 6% to 8% between 2008 and 2011, the increase most noticeable among 16- to 24-year-olds [14].

Since 2008, TTCs have been lobbying member states and the European Commission to remove the EU ban on snus sales [15]–[17], arguing that public health gains can be achieved if governments allow potentially reduced-risk products like snus on the market. The “Swedish Experience” is frequently cited as providing proof of concept that switching smokers from cigarettes to snus could be an effective harm reduction approach (Box 1). More recently BAT has argued that their investments in pure nicotine are driven by their ambition to provide smokers with a safer alternative to cigarettes [18],[19]. While many in public health support harm reduction [20], some suggest any such approach should be limited to nicotine rather than SLT products, while others raise concerns that reduced harm products may be promoted by TTCs for dual use with combusted products, which would ultimately be detrimental to public health [21].

**Box 1. The “Swedish Experience” Debate**

Swedish men have one of the lowest daily smoking rates in Europe [122], and one of the lowest rates of tobacco related disease (including lung cancer and cardiovascular disease) globally [123]. Many in public health attribute the high male snus consumption for the reductions in Swedish male smoking, proposing that this proof of concept could be replicated elsewhere in the EU and achieve net health gains [20],[124],[125]. Others [112],[126],[127], many from Scandinavia, have criticized this interpretation of Swedish data, countering that strong Swedish tobacco control measures instead played a significant role in reducing male smoking prevalence, highlighting that Swedish data show that only 5% of Swedish males smokers quit smoking using snus, that four out of 10 male snus users started their tobacco use with snus, and that almost as many continue to smoke and are dual users. Furthermore, they argue that smoking prevalence amongst Swedish women also significantly declined in the last 20 years (from 29% to 14%), albeit with no significant uptake in snus, thus indicating that snus is not associated with this decline.

Following significant controversy including the resignation of the European Health Commissioner [22], the European Commission recently, and belatedly, published proposals to revise the 2001 Tobacco Products Directive (TPD, 2001/37/EC) [23], the existing European legislation, which amongst other things, maintains the original 1992 ban on the sale of snus. Despite lobbying by industry and some public health groups, the European Commission proposes this ban on snus sales should be continued [24].

To inform this controversial policy debate, this paper aims to explore TTCs' interests in SLT and pure nicotine in Europe from the 1970s to the present. It examines TTC's historical interests, including efforts to enter European markets and influence national and EU public health policy, and the scale and nature of recent investments. The paper thus aims to compare the industry's privately documented interests (observed via internal documents and investor presentations) with those harm reduction pursuits it has publicly espoused, and to subsequently explore the implications for EU tobacco control policy.

## Methods

This study is based on qualitative analysis of internal tobacco industry documents, available on the online Legacy Tobacco Documents Library (http://legacy.library.ucsf.edu/) following litigation in the United States (US) [25]. Documents were retrieved from May 2010 to February 2011. Repeat searches in December 2012 identified no additional newer documents, likely due to the static nature of the tobacco document archive.

Most searches focused on BAT because preliminary searches had shown BAT to have been most active in Europe. We also included specific searches of Philip Morris (PM) and US Tobacco Company (UST) documents, the latter having collaborated with BAT to introduce SLT in Europe in the 1980s. Documents were initially identified and retrieved by SP via broad search terms (e.g. smokeless tobacco, snuff, snus, ST, OTP, innovative products, adjacency products), narrowed by using Boolean operators to include geographically specific search terms (e.g. Europe, EEC, EC, and EU). These initial searches were used to identify further search terms, including relevant project names (e.g. Penzance, Lotus, and Denver), internal committees (e.g. BAT's New Products Committee), and key personnel. Surrounding Bates numbers of key documents were also searched. The iterative process of searching, analysing, and refining by SP, overseen by AG, narrowed down over 15,000 documents to a final set of approximately 416 documents dating from 1971 to 2009. We were as comprehensive as possible in our searching and reached a point of document saturation, where new searches led to documents that had already been retrieved: an indicator that most important documents had already been identified. Analysis of these documents was based on an hermeneutic approach to company document analysis summarised by Forster [26], and complemented by the socio-historical archival techniques recommended by Hill [27] using an approach previously developed by AG [28]. This involves understanding the meaning of individual documents through reading and re-reading them over time and considering them alongside other documents, identifying themes and sub-themes and then triangulating the documents with other data. Where uncertainty over document meaning existed, these documents were reviewed by a second researcher. Finally, the documentary findings were placed in their broader context using other data sources including newspaper reports from the time [28].

A variety of data sources were used to triangulate the documentary findings. We compiled data specifically on TTC investments in snus and pure nicotine in the EU and systematically examined TTCs investor presentations, searching for the terms “harm reduction”, “reduced harm”, “smokeless”, and “snus”, and recording all occurrences. We limited this part of the analysis to those investor reports publicly available on the websites of PMI (www.pmi.com) and BAT (www.bat.com) in September 2012: 42 BAT presentations dating from 2007 to 2012 and 47 PMI presentations from 2008 to 2012. We also accessed BAT sustainability reports from 2001/02 (the first year of publication) to 2011 (prior to 2007 these were referred to as social reports), to identify further detail on BAT's snus investments. Furthermore, the paper draws on media reports on industry mergers, acquisitions and other developments identified via Nexus UK searches undertaken between January 2011 and January 2013 (using the names of the TTCs combined with terms “snus” and “smokeless” as search terms and no date limit), relevant tobacco industry journal (*Tobacco Reporter* and *Tobacco Journal International*) articles, Euromonitor reports, and other industry materials (notably press releases and websites).

## Results

### Internal Industry Documents

#### British American Tobacco in Western Europe

Despite TTC investment in SLT being a fairly new development, internal documents (with earliest BAT documents dating from 1971 and PM documents from 1978) reveal that BAT and PM have been investigating the concept for decades [29],[30]. Whereas PM primarily focused on scoping opportunities in the US, initially via acquisition and later via developing its own SLT product [31], during the 1970s and 1980s BAT actively explored opportunities in virgin SLT markets including South Africa, Australia, and Western Europe [32]–[34]. BAT's interest in SLT, prompted by an approach in 1971 from the American tobacco manufacturer UST (now US Smokeless Tobacco Company, subsidiary of Altria) [29], can be viewed in context of the TTCs' then operating environment. Revelations in the 1950s that smoking kills triggered substantial tobacco industry diversification, starting in the 1960s and continuing well into the 1980s [35],[36]. BAT for example invested in several businesses related to paper making, cosmetics, and food [36].

BAT's initial interest in diversifying into SLT in Western Europe arose from an awareness that health concerns about smoking would increase as would regulation, both threatening cigarette sales, and that SLT provided opportunities where smoking was prohibited [37]–[40]. This is illustrated in an internal briefing (1981) on SLT opportunities:

*We have no wish to aid or hasten any decline in cigarette smoking. Deeper involvement in smokeless is strategically defensible. There are fewer people in sophisticated markets starting to smoke. There are increasing numbers of people giving up. There are increasing restrictions on smoking, particularly in public, whether by law or by society* [32].

Table 1 further summarises themes we identified in four internal position papers examining BAT's arguments for and against investing in SLT and entering into a partnership with UST [32],[33],[41],[42]. Despite evidence that BAT scientists understood SLT to be “probably” less hazardous than smoking tobacco as early as 1971 [43], the potential health benefits of SLT over smoked tobacco products does not feature in these position papers, although SLT's ability to “provide a line of aggressive defence to the image and acceptability of tobacco and nicotine in general (and perhaps smoking indirectly)” [33] does (Table 1).

**Table 1:** Themes of BAT's rationales for and against investing in SLT, and partnering with UST, based on 1980s BAT documents.

	Pros	Cons
**Investment in SLT**	Fewer people starting smoking, more people quitting smokingNo passive smoking with SLT, thus more socially acceptableRestrictions on smoking in publicSLT can defend the image of the industry and its core product, nicotineSLT profit margins will be high (low tax and low production costs)Growth of SLT market in US, in particular moist snuff segmentCompetitors will capitalise on SLT opportunities if BAT doesn't act	There's a stigma to using SLTSLT is an acquired taste, thus initial sales in new markets will be labour- and cost-intensiveSuccess of SLT products in US due to freedom in advertising and promotionSome adverse health publicity in US and more attention likely if cigarette companies enter the businessSLT doesn't offer/substitute some of the socio-psychological pleasures of cigarettes
**UST partnership**	UST is the market leader in US, is very profitable, and enjoys a good reputation within the tobacco industryUST is pioneer in active marketing of SLTLimited costs for BAT; factories, existing products, brands, and trademarks in placeNo alternative; UST is only company that approached BATRumour that competitors are interested in UST	UST has limited product development and research facilitiesQuestionable product manufacturing quality and development facilities (which “in line with all US companies”, BAT found “lacking” [32])Only two UST trademarks experiencing growth in the US, and one in Canada

Based on four separate BAT marketing briefings in the 1980s [32],[33],[41],[42].

BAT's preliminary discussions with UST were abandoned in 1973 when BAT's concept evaluation (codename “Penzance”) exploring European consumer acceptance of SLT [34] came back with unsatisfactory results [44]. However, discussions between the two companies resumed in the early 1980s following BAT's realisation that, despite smoking prevalence and cigarette consumption falling in markets like the United Kingdom (UK) [45], tobacco remained its most profitable business (compared to its non-tobacco activities) [46]. Negotiations gained momentum when UST developed Skoal Bandits in 1983, a SLT “starter product” [47]. Skoal Bandits was a legacy of project “Lotus,” a former collaboration between UST and state-owned Swedish Tobacco (now Swedish Match, the largest European SLT manufacturer and market leader in Scandinavia) which pioneered *portioned* moist snuff to make SLT easier for people to consume, including people as young as 15 years of age [48],[49].

BAT estimated that Skoal Bandits would generate *new* profits in Western Europe rather than “cannibalise existing profits from cigarettes” [50], with Skoal Bandits anticipated to appeal to new generations of better-educated people no longer interested in taking up smoking. BAT's objective was “to market the range to younger, urban consumers as an alternative way to enjoy tobacco” [50]. A Dutch test market for Skoal Bandits was proposed; initially Amsterdam was considered because it had “a large youth and student population”, but Utrecht, “which also has a university”, was identified as potentially a better test market [50].

BAT eventually agreed to a German test market [51], but withdrew a year later due to reported high levels of nitrosamines in Skoal Bandits [52]. Documents suggest BAT was as much concerned about the risk of controversy and damage to its reputation as about the actual health risks from nitrosamines. An internal BAT briefing in April 1985 reported that:

*BATCF's R&D people are checking the product and all reputable sources of published research. It will then be a judgement call on the risk of forming a joint company with USTCo, and marketing Skoal Bandits. If the BARCLAYS controversy did not exist, the willingness to accept the risk would be higher* [53].

The “Barclays controversy” refers to BAT's public relations disaster in the early 1980s when the company had marketed its new Barclay cigarette as ultra-low tar, whereas in reality the cigarette design had deceived official tar-measuring machines and delivered a much higher tar yield than measured [54]. Consequently, BAT's competitors had taken BAT to court in several countries, including Germany where BAT settled out of court because “[the public dispute] had assumed proportions which would have ruined all further chances on the German market for the initially very successfully launched brand” [55]. Thus the concern about high levels of nitrosamine in Skoal Bandits, and the controversy it could have caused, effectively ended two decades of BAT scoping SLT opportunities in Europe.

#### Entry of Skoal Bandits to the European market

Despite BAT's withdrawal, UST aggressively marketed SLT in the mid-1980s as “the new way to enjoy tobacco” [56] in several European markets, including the UK [57],[58]. Despite a voluntary agreement to curb tobacco industry marketing and the UK government insisting that “the tobacco industry could be relied upon to act responsibly” [59], an internal BAT memo reported that UST was “working the Universities”, including paying students to promote Skoal Bandits to peers [60]. UST's marketing tactics in the UK mirrored those used in the US where it had been heavily criticised for aggressively targeting young people through its College Marketing Program [61]–[64].

In an attempt to secure government support and thwart regulation in the UK, UST commissioned scientific research that claimed there was no causal link between Skoal Bandits and cancer [65]–[67] and sought out professional parliamentary lobbying services [68]. In what became known as the “Cash for Questions” scandal, it was revealed that two UK Members of Parliament had actively promoted UST's interests to Health Ministers and the UK Parliament from 1985 to 1989 while enjoying hospitality from UST (Box 2). Despite UST's extensive lobbying efforts, in 1989 the UK Government introduced the *Oral Snuff (Safety) Regulations* banning the sales of certain oral tobacco including Skoal Bandits [69]. This had been prompted by a public outcry and well-organised public health campaign against Skoal Bandits [70]. Two years later, however, a successful appeal by UST in the British High Court [69] nullified the ban [71].

**Box 2. The “Cash for Questions” Affair**

The Cash for Questions Affair was a political scandal in the 1990s in the UK. The scandal came to light when *The Guardian* newspaper published an article in October 1994 claiming that professional parliamentary lobby firm Ian Greer Associates had bribed two Conservative Members of Parliament (MPs), Neil Hamilton and Tim Smith, to put forth parliamentary questions (at £2,000 a question) on behalf of then Harrods owner Mohamed Al-Fayed [128]. A subsequent public inquiry by the House of Commons Select Committee on Standards & Privileges in 1997 found that Hamilton, and another MP Michael Brown, had also been providing parliamentary services to UST from 1985 to 1989.

According to evidence in the Committee's report [68], the parliamentary support for UST had included the two MPs asking questions in Parliament and put forth Early Day Motions [129],[130]. The MPs had also lobbied government ministers on behalf of UST. For instance, following the UK Government's announcement in February 1988 that it intended to ban oral tobacco [131], Brown and Hamilton met several times with Health Ministers Clarke, Currie, and Mellor to oppose the legislation [132]–[134]. Kenneth Clarke, who later became Deputy Chairman and Director for BAT, recalled in a written statement to the Committee that he certainly remembered “being lobbied vigorously by Neil Hamilton, who was very indignant about the prohibition” [134].

Furthermore, evidence from the Committee's report found that both MPs had been paid £6,000 each by Ian Greer Associates for introducing the lobby firm to UST [129],[135]. Neither MP had added the payment to the Register of Members' Interests, nor declared it for tax purposes [136]. In addition, both MPs enjoyed hospitality from UST, including trips to the US which were not declared on the Register of Members' Interests [129],[130],[135]. During the inquiry, Brown and Hamilton both refuted claims that they were consultants of UST, claiming instead that their support for Skoal Bandits and its manufacturer came from their libertarian views that people should have the right to make decisions for themselves without interference from the State [129],[130].

#### TTC response to 1992 ban on oral tobacco

Meanwhile, European opposition to Skoal Bandits, and tobacco in general, was growing. Fear of Skoal Bandits being aggressively targeted to young people throughout Europe prompted the European Parliament to propose an EU-wide ban on “oral tobacco” sales in September 1987 [72],[73]. This was in line with the World Health Organization's (WHO) recommendation, published several months earlier, urging countries with no history of SLT use to pre-emptively ban SLT, thus preventing it from becoming a future public health problem [72]. Despite UST's successful challenge in the British High Court which built on its successful annulment of similar legislation in Ireland [74], the EU-wide ban was enacted in 1992 under the amended Labelling Directive (Table 2).

**Table 2:** EU Tobacco Control Directives specifically addressing SLT.

Directive Name/Year	Directive Number	Requirements
Labelling Directive (1992)	92/41/EEC	SLT to carry “causes cancer” health warningDefinition of oral tobacco: *“all products for oral use, except those intended to be smoked or chewed, made whole or partly of tobacco, in powder or particulate form or in any combination of these forms—particularly those presented in sachet portions or porous sachets—or in a form resembling a food product”*Ban on placing on the market of tobacco for oral use as defined above
Tobacco ProductsDirective (2001)	2001/37/EC	SLT health warning changed to: “This tobacco product can damage your health and is addictive”SLT health warning required to be on most visible surface of pack and cover at least 30% of packBan on snus sales maintained, but derogation for Sweden based on Article 151 of the Act of Accession of Austria, Finland, and Sweden

Adapted from http://ec.europa.eu/health/tobacco/policy/.

Between 1987 (when the SLT ban was proposed) and 1992 (when it was enacted), we found very few internal industry documents indicating industry opposition to the SLT ban. Compared to the TTCs' very active opposition to subsequent EU tobacco control Directives [75]–[77] and its recent lobbying to remove the ban [78]–[81], this suggests the TTCs did not actively oppose the 1992 snus sales ban. The absence of activity is also consistent with evidence that the industry's EU lobby was underdeveloped in the 1980s and early 1990s [75]. Furthermore, the TTCs then dominating the European tobacco market had no commercial interests in SLT in Europe. (At the time, Sweden was not part of the EU, and the interests of Swedish Match's predecessor, Swedish Tobacco, were predominantly confined to Sweden).

Nevertheless, the proposed ban prompted the establishment of ESTOC in 1989, co-founded by UST and Swedish Tobacco, “to promote understanding of the industry and its products and dialogue with retailers, the media, regulatory and/or advisory bodies” [82],[83]. Although we searched UST documents, no evidence was found to indicate that ESTOC and its members directly lobbied against the proposed Directive. Documents do, however, suggest that the Confederation of European Community Cigarette Manufacturers (CECCM) briefly lobbied members of the European Parliamentary Committee on Legal Affairs to scrutinise the legality of the Directive, arguing that Article 100A was an invalid legal basis [84]. It is doubtful that CECCM was specifically threatened by the draft Labelling Directive, nor the ban on SLT. Instead it is more plausible that CECCM wished to challenge the legal basis of EU tobacco control legislation in general, a tactic that has been central to industry efforts to derail all key tobacco control efforts in Europe [75],[76],[85].

### Contemporary Industry Interests in SLT, Other Reduced Risk Products, and Harm Reduction

#### TTC investments in SLT and pure nicotine products

**Figure 1:**
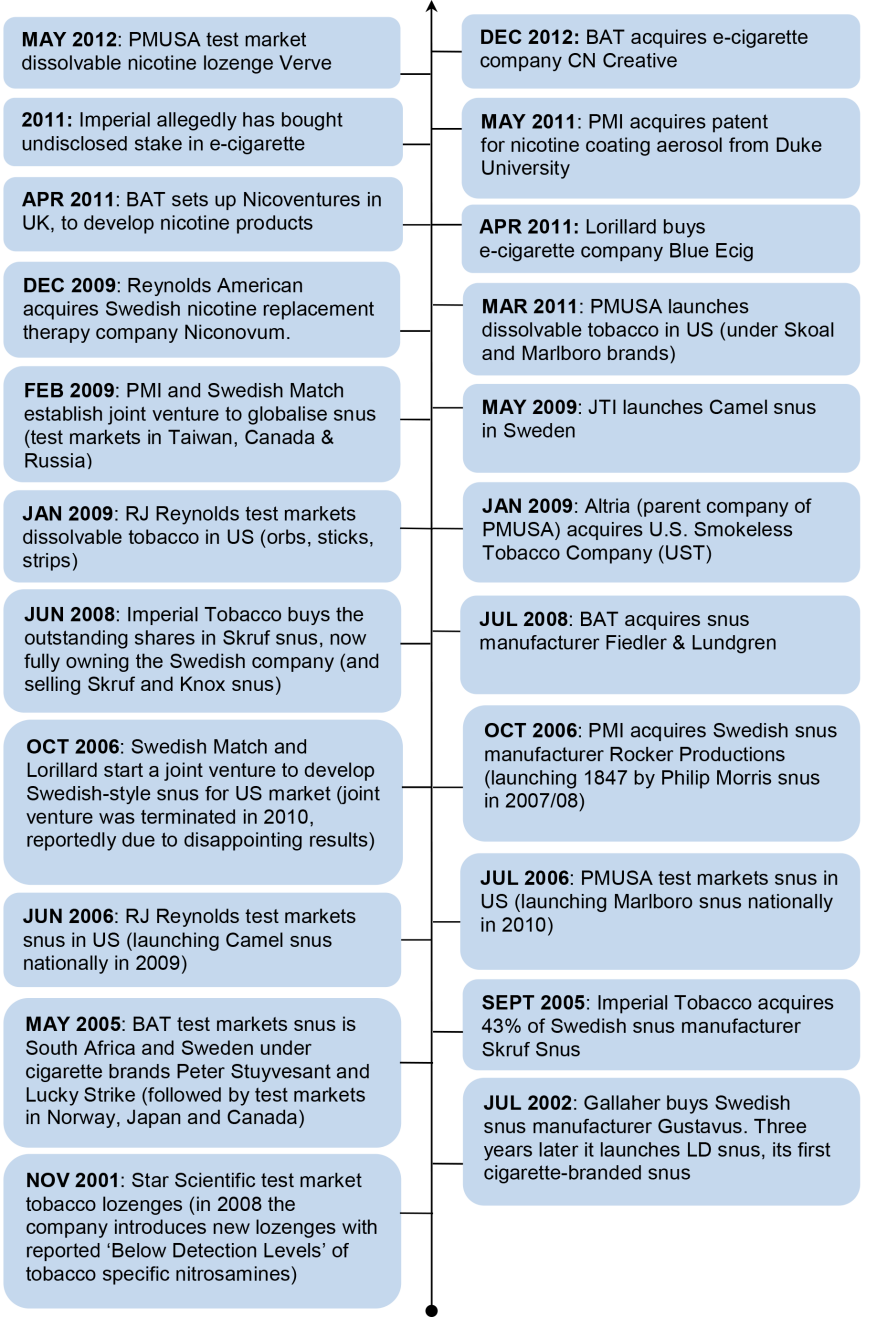
Timeline of TTC investment and activities in smokeless tobacco and nicotine markets. Source: media reports on industry mergers and acquisitions (identified via Nexus UK) and tobacco company websites.

Despite decades of scoping opportunities in SLT, no actual European investment took place until 2002, when Gallaher (now part of JTI) acquired snus manufacturer Gustavus. A flurry of other SLT investments followed, culminating in the PMI/Swedish Match joint venture in Feburary 2009 to sell snus outside Sweden and the US (Figure 1). While BAT claims its investment in snus occurred in response to consultation with public health experts [86], the timing of these investments in Europe also coincides with other notable developments. First, cigarette volumes in Western Europe declined from 2002 [4]. Second, discussions at EU level on smoke-free environments led to the 2003 EU Council recommendation (2003/54/EC) calling for Member States to provide protection from second-hand smoke in indoor workplaces, enclosed public places, and public transport [137]. Ireland and Norway became the first European countries to introduce smoke-free legislation in 2004, with all 27 EU Member States now having some form of smoke-free legislation in place [87]. The introduction of smoke-free legislation is reported to have significantly increased snus consumption in Sweden and Norway [88],[89], the former experiencing a 17% increase in snus sales volume in 2006, the year after Swedish smoke-free legislation was introduced [88]. Finally, TTC SLT investments also follow immediately from the first officially expressed high-level public health interest in tobacco harm reduction [90].

From December 2009 the focus of investment in reduced risk products switched from snus to pure nicotine products (Figure 1). Reynolds American's (of which BAT is the largest shareholder) acquired Swedish pharmaceutical company Niconovum in December 2009. In April 2011 BAT announced the establishment of Nicoventures to “commercialise non-tobacco nicotine products” [91] apparently investing £100 million [92], and in May PMI purchased a patent for a nicotine-containing aerosol [93]. In 2011 Imperial Tobacco reportedly purchased an “undisclosed stake” in an e-cigarette company [19] while in the US Lorillard announced in April 2012 that it had acquired e-cigarette company Blu Ecig [94], and Altria reported shortly thereafter that one of its subsidiaries was test marketing nicotine lozenges [95]. The most recent development is BAT's announcement that it has purchased e-cigarette company CN Creative [96].

#### Snus market shares in Europe

As a result of TTC investments in Swedish snus, all major snus companies have now either been acquired by, or entered into a joint venture with, TTCs [8]. Consequently, while Swedish Match retains the largest market share of the European (i.e., Scandinavian) snus market (Table 3), following the TTCs' investments this has declined by more than 10% in both Norway and Sweden over the last decade [97],[98]. Small, independent snus manufacturers account for only an insignificant proportion of the market (Table 3). Genuine competition between snus and cigarettes on the Scandinavian markets is thus slowly being reduced.

**Table 3:** Snus company market shares (volume) in Sweden and Norway by percentage, 2011.

Company	Sweden	Norway	Key Brands
**Swedish Match**Only listed EuropeanSLT manufacturer (sold its cigarette business in 1999)	85.7	69.6	General, Ettan, Kronan, Grovsnus, Göteborgs Rapé, Catch, Nick & Johnny, Lab series
**British American Tobacco**Acquired Fiedler & Lundgren	9.2	3.4	Granit, Mocca, Lucky Strike
**Imperial Tobacco**Acquired Skruf Snus	3.0	22.2	Knox, Skruf
**Japan Tobacco International**Acquired Gustavus snus when it took over Gallaher	2.0	0.0	Gustavus, LD, Camel
Others (small independent snus companies)	0.1	4.8	Thunder, Odens, Offroad, Jägerpris, Jakobsson's

Adapted from Euromonitor Passport GMID Sweden and Norway data [97],[98].

Snus brands from small independent companies were identified through random searches on the internet.

#### Contemporary TTC reporting on snus, pure nicotine and harm reduction

Only a few PMI and BAT investor presentations (2 out of 42 BAT presentations over the period 2007 to 2012 and 4 out of 47 PMI presentations 2008–2012) directly refer to their snus business, with PMI referring to snus as holding a “*long-term* promise in many markets” (emphasis added) [99]. The near absence of snus from BAT and PMI investor presentations suggests it is not a core part of their business strategy, a finding which could be explained by emerging evidence of limited value and volume growth opportunities in markets where SLT use is not already established. Although snus is cheap to produce, it is unclear what its profit margins are, certainly compared with the extraordinary profits for cigarettes [5],[100]. In a 2010 webcast to investors, PMI's then Chief Financial Officer, Hermann Waldemer, alluded to this when asked about PMI's joint venture with Swedish Match:

*It's something that will do us very good in the long term. This is why we went into this joint venture. However, short-term it doesn't have the same urgency and importance than it already has when it comes to the US market. The big profitability pools in the international tobacco world, often are, and continue to be, in the cigarette category. But you always need to be one step ahead, which is why we went into this joint venture* [101].

The volume growth potential for snus outside established SLT markets is arguably limited. Not only does growth in the EU require the snus sales ban to be lifted, but further afield snus test markets appear to have failed. In April 2011 BAT announced that it had “scaled back” snus test markets in Canada and South Africa, and was no longer selling snus outside Scandinavia [102], partly because “smokers often did not like using it in preference to cigarettes” and partly because BAT wasn't given regulatory support to market the product as reduced risk [103]. Furthermore, PMI recently remarked that, in regards to their snus test markets with Swedish Match, “As expected, initial consumer adoption is slow” [104].

Only very recently, following their investments in pure nicotine in 2011, have three investor presentations (one BAT [105] and two PMI [104],[106]) included details on harm reduction efforts, but then largely in the context of reduced risk products other than snus. Although both BAT's and PMI's presentations briefly acknowledged SLT and/or snus as an existing reduced risk product, BAT's David O'Reilly (Head of Research & Development) suggested that harm reduction and a “portfolio of commercially successful lower risk products” would lead to “revenue growth potential”, and, crucially, have “new ‘would be smokers’ begin with and stay with low risk product categories” [105]. PMI's most recent presentation, on the other hand, focussed on PMI's “Next Generation Products” (both tobacco and pure nicotine), of which it claimed that the first of three types will be on the market by 2017 [104].

## Discussion

### Key Findings

A number of important findings emerge from this paper. The documentary findings indicate that, historically, BAT's interest in SLT was driven purely by business concerns—the threat of regulation, particularly smoke-free regulation, and growing health concerns, both likely to result in falling cigarette sales. In this context SLT was seen as having the potential “to generate new profits without cannibalising existing profits from cigarettes” [50] by creating a *new* form of tobacco use among those that would no longer take up smoking due to health concerns. Yet concern about the health impacts of its products was not a rationale for investment and, despite BAT's scientists being aware, from at least 1971, that SLT was “probably” safer than smoked tobacco, BAT did not actually directly sell SLT until 2005. By contrast, BAT identified public relations opportunities emerging from SLT [33] and it was this reputational concern that ultimately prompted BAT to end its on–off association with UST in 1985 after high levels of nitrosamines were reported in Skoal Bandits. Overall, therefore, the documents suggest that BAT had little intention of promoting SLT use in a way that would encourage adult smokers to switch to SLT permanently as a means of reducing the risks of smoking, an approach now publicly espoused by the TTCs.

The documents also make it clear that young people were seen as the key target for SLT. Portioned snus was pioneered to make it easier for young people to use, and European test markets were identified on the basis of having large youth and student populations. Furthermore, when UST's Skoal Bandits was eventually launched in the UK, students were both the target and the means of promotion [60].

A number of findings suggest that the TTCs' current SLT strategy is very similar to its historical approach. First, the timing of the eventual TTC investments in snus is consistent with the original interest in SLT being driven by the dual threats of regulation and declining sales, alongside reputational opportunities. Having explored SLT throughout the 1970s and 1980s, the TTCs did not invest until 2002, when a flurry of investment activity followed. We show that these investments coincided with growing regulatory threats to cigarette sales including smoke-free legislation and with documented high level public health interest in harm reduction [90], the latter highlighting potential reputational benefits, and the former offering smokers temporary nicotine relief when smoking is prohibited.

Second, and perhaps most important, is that collectively our evidence suggests that TTC snus investments were defensive—by buying up snus manufacturers, the TTCs have turned snus from a threat (a product that may have competed with cigarettes) to a major opportunity (one that enables them to claim a joint agenda with public health and to ensure their long-term future should cigarette sales ultimately decline further or their profit margins be eroded). A number of factors support this argument. The TTCs have now bought up, or are in joint venture with, all significant snus manufacturers to the extent that the only manufacturers that fully remain independent of cigarette interests are tiny companies. Thus any genuine competition between snus and cigarettes has been eliminated; TTCs have ensured snus cannot cannibalise their highly profitable cigarette market and have increased their already considerable pricing power [100]. Yet despite these investments, we found little evidence that snus was a core part of BAT's or PMI's business strategy: it does not consistently feature in their investor reports, BAT has recently abandoned its snus test markets, and PMI's limited references to snus suggest it is of interest only in the long term [104] and that meanwhile the big profits continue to come from cigarettes [101]. Third, there is some suggestion from recent data that non-smokers, rather than smokers, would be the target of reduced risk products [105], entirely consistent with BAT's historical desire to create a new form of tobacco use to help overcome falling cigarette sales and reduced smoking uptake. This is also consistent with observations that in Russia (a virgin SLT market and snus test market of the PMI/Swedish Match joint venture), a snus marketing campaign appears to target young adults and non-tobacco users [107].

Another key finding in relation to the TTC's SLT interests is that both BAT's original and latest test markets appear to have failed, suggesting that snus may not work as a consumer product in virgin markets. Although there are few documents on BAT's 1973 exploration of consumer acceptance of SLT in Europe, it is clear the results were negative [44]. Similarly, BAT's test markets in Canada and South Africa appear to have failed and BAT is no longer selling snus outside Sweden (despite its ongoing rhetoric on harm reduction).

While we are less able to comment on the rationale for the TTC's very recent pure nicotine investments, it is likely that, like snus investments, they were prompted, like the snus investments, by recognition of both the potential reputational and political benefits and further concerns about the sustainability of the tobacco market. We note that industry analysts were questioning the long-term sustainability of the cigarette market [6] shortly before PMI and BAT's nicotine investments. Furthermore, the TTCs' investments in nicotine, on top of those in SLT, will have served to further reduce competition in the European nicotine market (which we define as encompassing all nicotine products, from cigarettes, the most harmful, to pure nicotine, the least harmful). This will enhance the already considerable profitability of cigarettes [100] and thus help ensure the long-term sustainability of the TTCs whose profits currently rely almost exclusively on cigarettes.

### Strengths and Limitations

One of the strengths of this paper is that it combines historical document research with analysis of contemporary industry materials, triangulating the documentary findings and overcoming the issue that most retrieved documents predate 2002. It is unique in examining industry interests in SLT in Europe, complementing findings from the US (an established SLT market) that SLT is marketed to augment cigarette use and offset smoke-free regulations [31],[108]–[111].

The nature of tobacco industry document research means that we made decisions about relevant search terms and document inclusion. Inadvertently, this may have led to relevant documents being omitted. Furthermore, our analysis is limited to documents made public following litigation; these document collections may not be fully representative of all documents within the corporations subject to the litigation and do not cover companies (Imperial Tobacco, JTI, or Swedish Match) not subject to the litigation, limiting our ability to explore their historical interests in this area. Consequently, and because the documents indicated that PM's historical interest in SLT was in the US, our early findings relate to BAT and UST. It is possible therefore that the absence of documents indicating sustained industry opposition to the 1992 ban on snus sales reflect a weakness in the document collections rather than a genuine absence of industry activity. However, we think this unlikely given that extensive searches were undertaken of the UST documents on this issue and previous evidence indicates that the EU tobacco lobby was underdeveloped in that time [75].

### Policy Implications

Our findings have a number of implications for public health policy and highlight the complexity of the debates around snus. First, they indicate that the industry's rhetoric on harm reduction has been inconsistent with historical and recent documents and business actions. Instead, the findings suggest that the TTCs' interest in reduced-risk products lies in maintaining the status quo in favour of cigarettes for as long as possible while simultaneously providing a longer-term source of profit should the cigarette model prove unsustainable; the reputational benefits are an additional asset. The fact that SLT investments in Europe coincided with the implementation of smoke-free policies, combined with evidence of the industry's promotion of dual cigarette and snus use in the US [31],[108]–[111], adds weight to the concern that TTCs may hope to exploit snus as a way to reduce the impact of regulations aimed at reducing smoking rates.

Second, a number of findings suggest that the generalisability of the “Swedish experience” to countries in which SLT is not traditionally used may be limited: the failure of snus test markets; the lack of competition between cigarettes and snus which is a new phenomenon and remains greater outside Sweden; and evidence that the industry's historic interest in snus was both because it could be used in smoke-free environments and could be promoted to young, non-tobacco users to create a new form of tobacco use. This last finding lends support to concerns that SLT may lead to, rather than from, smoking [111]–[113]. Further evidence that the Swedish experience may not be generalizable comes from a recent study concluding that even in a market with a history of SLT use like the US, smokers did not consider snus an acceptable substitute for smoking or a way to quit smoking [114].

While such evidence must be considered alongside the broader body of evidence around snus and the fact it is significantly less harmful than smoked tobacco [115]–[117], collectively these issues suggest that legalising snus sales in Europe may have considerably less benefit than envisaged and could have a number of harmful consequences. Perhaps of greater concern, however, given that harm reduction using nicotine products is already an established element of tobacco control [118] and recent research suggests scope for benefit via newer nicotine products [119], are the recent industry investments in pure nicotine products. These raise two concerns. First, one of competition: should such investments continue, competition between cigarettes and clean nicotine products would decrease, limiting the potential for harm reduction to benefit public health and maintaining the status quo of cigarettes. While a nicotine regulatory authority could ensure that regulation was proportional to harm [20],[120], it would be powerless to address the issue of competition, so this situation needs close observation. Second, they may enable TTCs, by presenting themselves as purveyors of nicotine rather than tobacco products, to undermine Article 5.3 of the Framework Convention on Tobacco Control [121] which aims to protect public health policy from commercial and other vested interests of the tobacco industry. Finally, if TTCs are genuinely interested in seeing their cigarette consumers switch to snus (or pure nicotine products), rather than creating new snus/nicotine users and/or dual use opportunities, we would expect to see detailed strategic plans and cigarette sales reduction targets at least for the markets where they intend to introduce these products. However, to this date we have yet to see this.

## Abbreviations

BAT: British American Tobacco
CECCM: European Community Cigarette Manufacturers
ESTOC: European Smokeless Tobacco Council
EU: European Union
JTI: Japan Tobacco International
MP: Member of Parliament (UK)
PM: Philip Morris
PMI: Philip Morris International
SLT: smokeless tobacco
TPD: Tobacco Products Directive
TTC: Transnational Tobacco Company
UST: US Tobacco Company
